# Shorter dialysis session length was not associated with lower mental health and physical functioning in elderly hemodialysis patients: Results from the Japan Dialysis Outcome and Practice Patterns Study (J-DOPPS)

**DOI:** 10.1371/journal.pone.0184019

**Published:** 2017-09-06

**Authors:** Masashi Kitagawa, Ken-ei Sada, Norikazu Hinamoto, Miho Kimachi, Yosuke Yamamoto, Yoshihiro Onishi, Shunichi Fukuhara

**Affiliations:** 1 Department of Nephrology, Rheumatology, Endocrinology and Metabolism, Okayama University Graduate School of Medicine, Dentistry and Pharmaceutical Sciences, Okayama, Okayama, Japan; 2 Department of Healthcare Epidemiology, School of Public Health in the Graduate School of Medicine, Kyoto University, Kyoto, Kyoto, Japan; 3 Institute for Health Outcomes and Process Evaluation Research (iHope International), Kyoto, Kyoto, Japan; 4 Center for Innovative Research for Communities and Clinical Excellence, Fukushima Medical University, Fukushima, Fukushima, Japan; Postgraduate Medical Institute, INDIA

## Abstract

**Background:**

Health-related quality of life (HRQOL) is often prioritized over long-term survival in elderly patients. Although a longer dialysis session length (DSL) has been shown to reduce mortality, its effects on improving the HRQOL are unknown.

**Methods:**

Using data from the Japan Dialysis Outcomes and Practice Patterns Study (J-DOPPS), patients aged ≥ 65 years on maintenance hemodialysis were enrolled. DSL was categorized as short (<210 minutes), medium (210–240 minutes), or long (>240 minutes). The primary outcomes were changes in mental health (ΔMH) and physical functioning (ΔPF) scores assessed using the Japanese version of SF-12, in one year. The differences in the ΔMH and ΔPF among the three groups were assessed via regression (beta) coefficients derived using a linear regression model.

**Results:**

Of 1,187 patients at baseline, 319 (26.9%) had a short length, 686 (57.8%) a medium length, and 182 (15.3%) a long length. We assessed the ΔMH data from 793 patients and the ΔPF data from 738. No significant differences in the ΔMH were noted for the short or long groups compared with the medium group (score difference: 0.26, 95% confidence interval [CI]: -4.17 to 4.69 for short; score difference: -1.15, 95% CI: -6.17 to 3.86 for long). Similarly, no significant differences were noted for these groups versus the medium group in ΔPF either (score difference: -1.43, 95% CI: -6.73 to 3.87 for short; score difference: -1.71, 95% CI: -7.63 to 4.22 for long).

**Conclusions:**

A shorter DSL might have no adverse effects on MH or PF for elderly patients.

## Introduction

The mortality rates of hemodialysis (HD) patients continue to improve gradually, and Japanese HD patients in particular have the lowest mortality rates in the world [1]. With these improving mortality rates, the health-related quality of life (HRQOL) is gaining increasing importance as a relevant outcome [2]. However, the HRQOL in HD patients remains relatively low compared to that in the general population [3].

Among elderly HD patients, HRQOL is often prioritized over long-term survival. Although many previous reports have indicated that a longer dialysis session length is associated with a reduced mortality [4–9], dialysis session length in daily clinical practice is shortened in elderly patients for any of several reasons, such as reducing the physical burden. Indeed, the mean dialysis session length is shorter in elderly patients than in overall HD patients in Japan (231 minutes in elderly patients vs. 241 minutes in total HD patients; data provided by the Japanese Society for Dialysis Therapy) as well as in other countries [10, 11]. Therefore, the dialysis session length in elderly patients may be shortened not with respect to mortality but based on other determinants, such as HRQOL, provided the dialysis efficiency was sufficient.

Previous studies have reported that dialysis session length and treatment interval were associated with mental health (MH) and physical functioning (PF), but these findings are controversial [12–17]. Considering these results, the dialysis session length may ameliorate MH and PF through the improvement of dialysis efficiency, whereas the long restriction required for longer-duration treatment may deteriorate MH and PF in conventional HD patients. Therefore, the association of the MH and PF with dialysis session length in elderly patients needs to be examined.

Here, we evaluated the influence of the dialysis session length on the MH and PF in elderly HD patients as indicators of HRQOL. We hope these findings will guide clinicians on the optimum dialysis session length based not only on mortality but also HRQOL.

## Materials and methods

### Design, setting, and participants

We enrolled hemodialysis patients who participated in the third (2005–2008) and fourth (2009–2011) phase of the Japan Dialysis Outcomes and Practice Patterns Study (J-DOPPS). The design of the DOPPS is detailed in a previous report [18].

A total of 121 facilities were included in J-DOPPS phases 3 to 4. For this study, eligible participants were patients aged 65 years or older on maintenance HD for at least 120 days. We excluded participants with missing data on dialysis session length, MH and PF subdomain scores in the Japanese version of the SF-12, and vintage. J-DOPPS data, which included laboratory data, drug information, and dialysis conditions, were collected every four months. Our present study using these J-DOPPS data complied with the Declaration of Helsinki. All participants in J-DOPPS have provided written informed consent before study enrollment. Data collection was performed in a fashion that maintains patient anonymous at the cording center. This study’s conduct was approved by the Ethics Committee of Tokyo Women's Medical University (Approval Numbers 709, 1178, 1278, 1527, 1826, and 2143).

### Main exposure

The main exposure was dialysis session length at baseline, categorized into three groups: (1) short length (≤ 210 minutes per session), (2) medium length as reference (210–240 minutes per session), and (3) long length (>240 minutes per session). In Japan, over half HD patients is prescribed 240 minutes dialysis session length and dialysis session length for the great majority of patients is at exactly 30 min intervals [11, 19, 20]. Therefore, we set the medium length group under the assumption of 240 minutes group as reference.

### Main outcome

The primary outcomes were changes in mental health (ΔMH) and physical functioning (ΔPF), as assessed using the Japanese version of the SF-12 [21], at one year after study initiation.

The subdomain of MH was composed of the following questions:

1. Have you felt calm and peaceful?
2. Have you felt downhearted and blue?

The subdomain of PF was composed of the following questions:

Does your health now limit you in these activities? If so, how much?

1. Moderate activities, such as moving a table, pushing a vacuum cleaner, bowling, or playing golf
2. Climbing several flights of stairs

The scores of each subdomain were totaled and transformed to a scale of 0–100 points, with higher scores representing better health states.

### Statistical analyses

Baseline characteristics of participants were categorized by dialysis session length and described respectively. Characteristics of continuous data with normal distribution were summarized as means (±standard deviation [SD]), continuous variables with skewed data as medians (inter-quartile range [IQR]), and dichotomous or categorical data as proportions.

We compared the unadjusted and multivariable-adjusted score differences to evaluate the association between each duration on HD and ΔMH or ΔPF at one year after study initiation.

The model was adjusted for potential confounders at baseline, including age, gender, smoking habit, dialysis vintage, calcium level, albumin level (Alb), ultrafiltration rates (UFR), presence of comorbidities (diabetes, cardiovascular disease, congestive heart failure, dysrhythmia, other cardiac diseases, cerebrovascular disease, peripheral vascular disease, gastrointestinal bleeding, liver disease, cancer, chronic obstructive pulmonary disease, and chronic hepatitis), soporific or antidepressant drugs (benzodiazepines, selective serotonin reuptake inhibitors, serotonin antagonist and reuptake inhibitors, selective melatonin receptors, noradrenergic and specific serotonergic antidepressant, tetracyclic antidepressants) and erythropoietin resistance index (ERI). The severity of comorbidities were presented as their Charlson Index, ranging from 0 to 21 [22]. The ERI was evaluated by dividing the average weekly erythropoiesis-stimulating agent dose by body weight (kg) per average hemoglobin value (g per dL). A diagnosis of depression was defined as Center for Epidemiologic Studies Depression Scale (CES-D) < 10%. Linear mixed effect models were developed to assess the score differences in ΔMH and ΔPF, taking into account cluster effects at the facility level.

Additionally, we performed multiple imputation using predictive mean matching (pmm) for complementation of missing covariates. The missing data were imputed 10 times to obtain 10 complete datasets. We then used Rubin’s rule with summary estimates to combine the results from multiple imputed datasets.

Further, as a sensitivity analysis, we assessed the influence of missing data for MH or PF in one year. We also performed multiple imputation using pmm with 10 repetitions for complementation of missing outcomes for participants censored for reasons other than death.

All statistical analyses were performed using STATA 14.0 (version 14.0; StataCorp, College Station, TX, USA), with 2-sided significance set at 0.05.

## Results

### Study flow diagram

**Fig 1:**
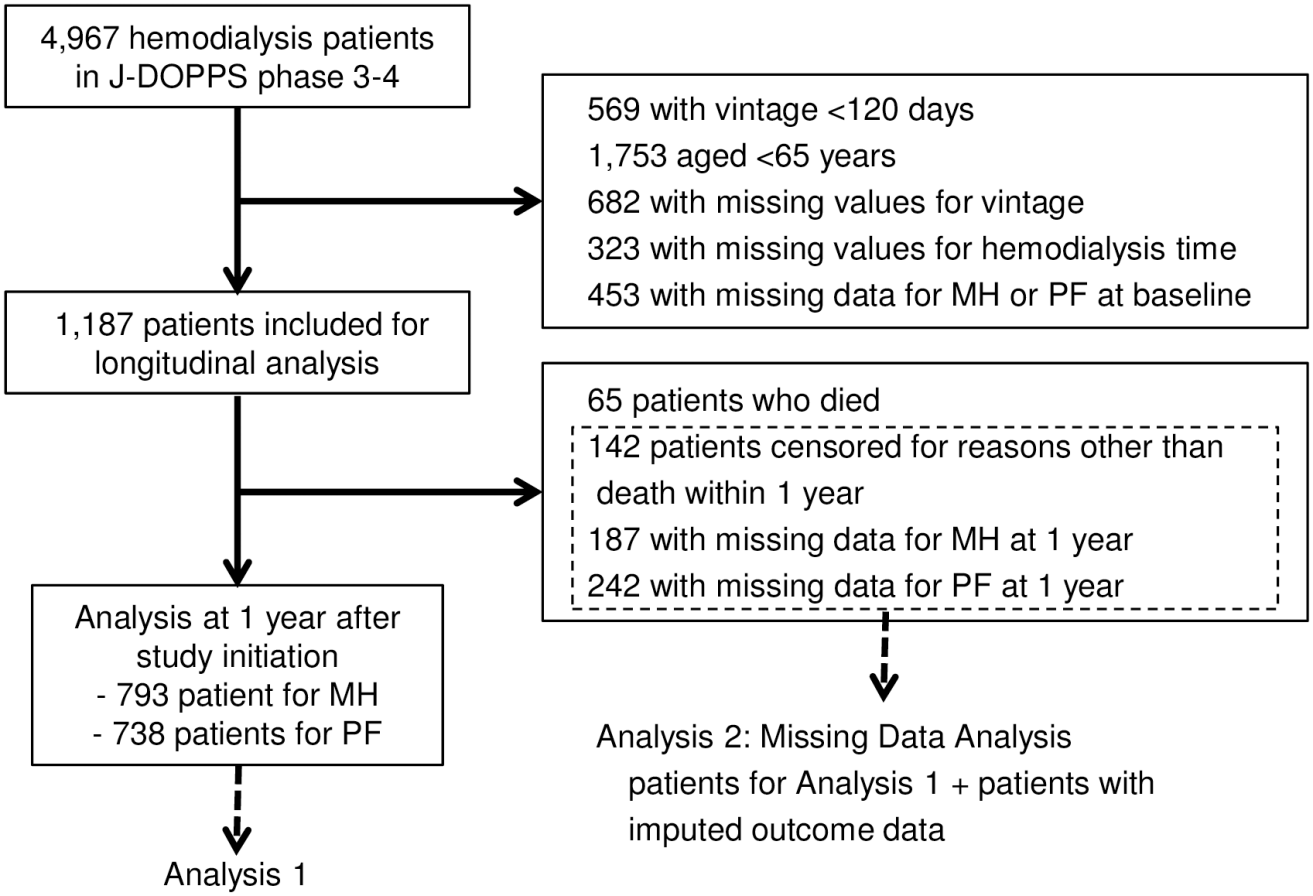
Participant flow diagram and the study selection process.

In accordance with pre-defined criteria, 1,187 patients were included in this study. After several dropouts, for reasons such as death, censoring other than death, and missing values of MH and PF at 1 year, 793 and 738 patients remained in the analysis of the score difference of MH and of PF at 1 year, respectively (Fig 1).

### Baseline characteristics of study participants

Dialysis session length showed a normal distribution, and the overall mean was 231.1 (29.4) minutes per session at baseline. With regard to each category, 319 (27.6%) subjects were categorized as short session length, 686 (57.8%) as medium, and 182 (15.3%) as long.

Table 1 shows the baseline characteristics of patients, categorized by time on HD per day. Mean age was 73.2 years (range 65 to 94 years), 62.9% of subjects were male, the median dialysis vintage was 6.6 years, and the mean Charlson Index was 4.1. Patients with longer time were more likely to be male and have a smoking habit, longer vintage, and lower ERI. Patients with shorter times were more likely to have lower MH and PF than those with a medium session length.

**Table 1:** Baseline characteristics of patients, categorized by dialysis session length.

Characteristics	Total (n = 1,187)	Time≤210 (n = 319)	210<Time≤ 240 (n = 686)	240<Time (n = 182)
Age, years	73.2 (6.3)	74.7 (6.9)	72.6 (6.0)	72.4 (5.9)
Male, %	62.9	58.2	62.8	71.4
Time on hemodialysis, minutes	231.1 (29.4)	191.1 (16.1)	239.7 (2.4)	268.8 (25.6)
Dialysis time per week, times	3.0 (0.20)	2.9 (0.33)	3.0 (0.11)	3.0 (0.13)
Smoker (ever), %	36.9	34.9	34.3	48.3
Vintage, years	6.6 (6.5)	3.2 (3.4)	7.8 (6.8)	8.3 (7.3)
Charlson Index	4.1 (3.5)	4.0 (3.4)	4.3 (3.6)	3.9 (3.4)
CES-D < 10, %	50.3	45.1	53.8	45.5
Hemoglobin, g/dL	10.3 (1.2)	10.2 (1.2)	10.3 (1.2)	10.6 (1.2)
Calcium, mg/dL	9.2 (0.79)	9.1 (0.81)	9.2 (0.77)	9.4 (0.78)
Albumin, g/dL	3.7 (0.37)	3.7 (0.44)	3.7 (0.35)	3.7 (0.35)
Ultrafiltration Rates, ml/mmHg/h	9.9 (5.5)	10.5 (5.4)	9.6 (5.4)	10.3 (6.1)
ERI, U/week/kg/g/dL	8.6 (7.7)	9.0 (6.8)	8.8 (8.2)	7.5 (7.1)
Soporific, N (%)	29.2	28.2	29.3	30.8
Anti-depression medicine, N (%)	3.4	2.2	4.2	2.2
Mental health	64.0 (23.9)	61.1 (25.2)	65.5 (23.2)	63.1 (24.0)
Physical functioning	50.1 (34.4)	45.1 (33.5)	52.1 (34.2)	51.6 (35.9)

Continuous data with a normal distribution are summarized as the mean (standard deviation), continuous variables with skewed data are summarized as the median (interquartile range), and dichotomous or categorical data are summarized as proportions.

### Changes in MH

At 1 year after study initiation, the ΔMH of the 793 subjects (Fig 1, Analysis 1) was -1.42 (24.6). By category, the ΔMH in the short session length group was -0.35 (23.8), that in the medium session length group was -1.5 (25.4), and that in the long session length group was -2.5 (22.3).

As shown in Table 2, a short session length had no significant influence on the ΔMH versus a medium length as a reference during the 1-year observational period (score difference: 0.26, 95% confidence interval [CI]: -4.17 to 4.69, and p value: 0.91), nor did a long session length (score difference: -1.15, 95% CI: -6.17 to 3.86, and p value: 0.65).

**Table 2:** Association between dialysis session length and change in mental health or physical functioning at one year after study initiation.

	Unadjusted β	Adjusted β
**Mental Health**		
Time≤210	1.21 (-3.04 to 5.47)	0.26 (-4.17 to 4.69)
210<Time≤ 240	Reference	Reference
240<Time	-0.97 (-5.95 to 4.02)	-1.15 (-6.17 to 3.86)
**Physical Functioning**		
Time≤210	-2.37 (- 7.41 to 2.68)	-1.43 (-6.73 to 3.87)
210<Time≤240	Reference	Reference
240<Time	-1.32 (-7.19 to 4.55)	-1.71 (-7.63 to 4.22)

Results shown are coefficients (95% confidence intervals).

Adjusted for age, gender, HD duration, smoking, Ca, Alb, UFR, Charlson index, ERI, and medication (soporific, antidepressant). Additionally, dialysis session length was not associated with ΔMH among all-aged participants of this study (S1 Table).

### Changes in PF

At 1 year after study initiation, the ΔPF of the 738 patients (Fig 1, Analysis 1) was -1.9 (28.1). By category, the ΔPF in the short session length group was -3.6 (28.3), that in the medium session length group was -1.1 (28.2), and that in the long session length group was -2.7 (27.7).

As found for MH, in Table 2, a short session length had no significant influence on the ΔPF versus a medium length as a reference during the 1-year observational period (score difference: -1.43, 95% CI: -6.73 to 3.87, and p value: 0.60), nor did a long session length (score difference: -1.71, 95% CI: -7.63 to 4.22, and p value: 0.57). Additionally, dialysis session length was not associated with ΔPF among all-aged participants of this study (S1 Table).

### Sensitivity analysis of missing data

Of the 1,187 patients at baseline, about 40% dropped out within 1 year from study initiation. Sixty-five patients were censored due to death (20 patients with short session length [6.3% of the short session length group], 33 patients with medium session length [4.8% of the group], and 12 patients with long session length [6.6% of the group]), 142 were censored for reasons other than death, and 277 had missing values for MH or PF at 1 year (187 patients with missing data for MH and 242 patients with missing data for PF). We performed multiple imputation using pmm for 1,122 participants with missing values of MH or PF due to reasons other than death (Fig 1, Analysis 2). The findings were consistent with the original results, and we confirmed that the missing data didn’t affect the overall study results.

## Discussion

This is the first report to investigate the relationships between the dialysis session length and MH and PF as indicators of HRQOL, which is a particularly relevant outcome for elderly patients. Dialysis session length was not significantly associated with MH or PF in the present study. We used a subdomain of the SF-12 to measure MH and PF. The SF-12 is constructed from the SF-36 and vigorously and concisely predicts the SF-36 Physical and Mental Component Summary scores [23–26]. Further, ΔMH and ΔPF have been evaluated as predictors of mortality in previous studies [26, 27].

In this study, aged HD patients with short dialysis sessions were more likely to be female and have a higher ERI. A previous study similarly reported that a shorter dialysis session was associated with older age and a higher prevalence of female patients [10, 11]. Given that other studies have reported an association between a shorter dialysis session and a higher ultrafiltration rate, a short dialysis session may be sufficient in elderly female patients because of their overall smaller stature than male patients [11]. However, ERI, which is associated with mortality, is known to be improved by increased intensity of dialysis including a longer dialysis session [28]. Taken together, these present and previous findings indicate that patients unlikely to survive long-term might receive a shorter dialysis session.

No session length-dependent factors influencing the ΔMH in aged HD patients were noted in the present study. Although only one cross sectional study reported no association between dialysis session length and MH in conventional HD patients [29], the ΔMH versus the dialysis session length has never before been evaluated. While previous reports have shown that daily short-session dialysis tended to improve the MH [12, 13], the effects of daily long-session dialysis are controversial [30]. In the present study, dialysis session length did not influence the ΔMH in elderly conventional HD patients. Therefore, in terms of MH, a short session length might be the better option in elderly patients, as these patients are more likely to have back pain and experience a burden due to inactivity than younger patients.

We also noted no association between dialysis session length and ΔPF. The above-referenced cross-sectional study reported an association between short dialysis session length and better PF in conventional HD patients [29]. However, a randomized controlled trial revealed that daily short-session dialysis did not improve the objective PF but did improve the subjective PF, while daily long-session dialysis did not improve either the objective or subjective PF [17]. As with MH, a short dialysis session length may not adversely affect the PF in elderly HD patients. Additionally, dialysis session length were not associated with both ΔMH and ΔPF among all-aged participants of this study (S1 Table). Further, we considered the influence of missing values of both ΔMH and ΔPF through sensitivity analyses. Although the point estimates of the coefficients were slightly deviated, we concluded that the differences in the ΔMH and ΔPF among the HD session length groups did not meet the minimum clinically important difference in comparison with the original results [31–33] (Table 3), and the findings were essentially the same as those obtained in the primary analyses.

**Table 3:** Multiple imputation using predictive mean matching (pmm) for censoring during study follow-up.

	Unadjusted β	Adjusted β
**Mental Health**		
Time≤210	1.75 (-2.28 to 5.78)	0.96 (-3.15 to 5.08)
210<Time≤ 240	Reference	Reference
240<Time	-0.52 (-5.48 to 4.44)	-0.67 (-5.67 to 4.34)
**Physical Functioning**		
Time≤210	-1.04 (-5.72 to 3.63)	-0.14 (-5.16 to 4.88)
210<Time≤240	Reference	Reference
240<Time	-0.71 (-6.47 to 5.05)	-0.51 (-6.23 to 5.20)

Results shown are coefficients (95% confidence intervals).

Adjusted for age, gender, HD duration, smoking, Ca, Alb, UFR, Charlson index, ERI, and medication (soporific, antidepressant).

Several limitations to the present study warrant mention. First, we conducted our evaluation using the dialysis session length at baseline. Dialysis session length can often fluctuate, but we did not evaluate the influence of such fluctuation during the observational period. Second, the model in this study wasn’t adjusted for unmeasured confounding factors, such as treatment principles depended on physicians, residual renal function, residual urine output, or urea clearance, because of the observational nature of the study. These limitations may have obscured the influence of dialysis session length on MH and PF. Third, our study excluded patients who could not respond to the questionnaire for MH or PF due to physical or intellectual disability. Baseline characteristics among participants with missing values of MH or PF were similar to the total means of those without missing outcomes (S1 Table), but any interpretation of the findings should be conducted with caution. Fourth, the SF-12 was validated using Japanese representative samples, and also validated internationally in dialysis patients [24, 34]. We can calculate norm-based scores based on the study. Actually, there are many reports using the SF-12 in Japanese patients with other various diseases [35–39]. In the recent report using DOPPS data, the SF-12 is used for measurement of health-related quality of life including Japanese HD patients [40].

In conclusion, the present results suggest that a shorter dialysis session length had no adverse effects on MH or PF in elderly patients.

## Supporting information

S1 Table Association between dialysis session length and ΔMH or ΔPF at one year after study initiation among all-aged participants. Results shown are coefficients (95% confidence intervals). Adjusted for age, gender, HD duration, smoking, Ca, Alb, UFR, Charlson index, ERI, and medication (soporific, antidepressant). Of 2,610 all aged participants, 1,882 patients had the results of ΔMH and 1,725 patients had the results of ΔPF.
(DOCX)
