# Supplementary material for: Shorter dialysis session length was not associated with lower mental health and physical functioning in elderly hemodialysis patients: Results from the Japan Dialysis Outcome and Practice Patterns Study (J-DOPPS)
Source: PLoS One. 2017 Sep 6;12(9):e0184019. doi: 10.1371/journal.pone.0184019 (PMC5587338; doi:10.1371/journal.pone.0184019)
Supplement: S1 Table — Results shown are coefficients (95% confidence intervals). Adjusted for age, gender, HD duration, smoking, Ca, Alb, UFR, Charlson index, ERI, and medication (soporific, antidepressant). Of 2,610 all aged participants, 1,882 patients had the results of ΔMH and 1,725 patients had the results of ΔPF. (DOCX) [file pone.0184019.s001.docx]

**S1 Table. Association between dialysis session length and change in mental health or physical functioning at one year after study initiation among all-aged participants**

|  | **Unadjusted β** | **Adjusted β** |
| --- | --- | --- |
| **Mental Health** |  |  |
| Time≤210 | 2.50 (-0.33 to 5.32) | 2.38 (-0.56 to 5.31) |
| 210<Time≤ 240 | Reference | Reference |
| 240<Time | 1.53 (-1.13 to 4.20) | 1.24 (-1.49 to 3.97) |
| **Physical Functioning** |  |  |
| Time≤210 | -1.01 (-4.43 to 2.42) | -0.17 (-3.73 to 3.38) |
| 210<Time≤240 | Reference | Reference |
| 240<Time | 1.60 (-1.65 to 4.84) | 0.99 (-2.32 to 4.31) |

Results shown are coefficients (95% confidence intervals). Adjusted for age, gender, HD duration, smoking, Ca, Alb, UFR, Charlson index, ERI, and medication (soporific, antidepressant). Of 2,610 all aged participants, 1,882 patients had the results of ΔMH and 1,725 patients had the results of ΔPF.
